# Acceptability of integrating smoking cessation treatment into routine care for people with mental illness: A qualitative study

**DOI:** 10.1111/hex.13580

**Published:** 2022-10-12

**Authors:** Katherine Sawyer, Kim Fredman Stein, Pamela Jacobsen, Tom P. Freeman, Anna K. M. Blackwell, Chris Metcalfe, David Kessler, Marcus R. Munafò, Paul Aveyard, Gemma M. J. Taylor

**Affiliations:** ^1^ Department of Psychology, Addiction and Mental Health Group University of Bath Bath UK; ^2^ Department of Psychology, Addiction and Mental Health Group, Bath Centre for Mindfulness and Compassion University of Bath Bath UK; ^3^ Bristol Randomised Trials Collaboration, Population Health Sciences Bristol Medical School Bristol UK; ^4^ Department of Population Health Sciences, Bristol Medical School, Centre for Academic Primary Care University of Bristol Bristol UK; ^5^ MRC Integrative Epidemiology Unit, School of Psychological Science University of Bristol Bristol UK; ^6^ Nuffield Department of Primary Care Health Sciences University of Oxford Oxford UK

**Keywords:** anxiety, depression, IAPT, primary health care, qualitative, smoking cessation

## Abstract

**Introduction:**

Improving Access to Psychological Therapies (IAPTs) Services could offer smoking cessation treatment to improve physical and psychological outcomes for service users, but it currently does not. This study aimed to understand participants' views and experiences of receiving a novel smoking cessation intervention as part of the ESCAPE trial (intEgrating Smoking Cessation treatment As part of usual Psychological care for dEpression and anxiety). We used the Capability, Opportunity and Motivation Model of Behaviour (COM‐B) to understand the (i) acceptability of the integrated smoking cessation treatment, (ii) views of psychological well‐being practitioners' (PWPs) ability to deliver the smoking cessation treatment and (iii) positive and negative impacts of smoking cessation treatment.

**Methods:**

This was a qualitative study embedded within a feasibility randomized‐controlled trial (ESCAPE) in primary care services in the United Kingdom (IAPT). Thirty‐six participants (53% female) from both usual care and intervention arms of the ESCAPE trial, including both quitters and nonquitters, were interviewed using semi‐structured interviews. Data were analysed using a framework approach to thematic analysis, using the COM‐B as a theoretical frame.

**Results:**

Psychological Capability: Integrated smoking cessation treatment was acceptable and encouraged participants to reflect on their mental health. Some participants found it difficult to understand nicotine withdrawal symptoms. Motivation: Participants were open to change during the event of presenting to IAPT. Some described being motivated to take part in the intervention by curiosity, to see whether quitting smoking would help their mental health. Physical Opportunity: IAPT has a natural infrastructure for supporting integrated treatment, but there were some barriers such as session duration and interventions feeling segmented. Social Opportunity: Participants viewed PWPs as having good interpersonal skills to deliver a smoking cessation intervention.

**Conclusion:**

People with common mental illness generally accepted integrated smoking cessation and mental health treatment. Smoking cessation treatment fits well within IAPT's structure; however, there are barriers to implementation.

**Patient or Public Contribution:**

Before data collection, we consulted with people with lived experience of smoking and/or mental illness and lay public members regarding the aims, design and interview schedules. After analysis, two people with lived experience of smoking and mental illness individually gave feedback on the final themes and quotes.

## 1 INTRODUCTION

Smoking is the world's leading cause of cancer and death worldwide.^1^, ^2^, ^3^ People with common mental illness, such as depression and anxiety, are twice as likely to smoke than those without common mental illness. In the United Kingdom, smoking prevalence in people with depression or anxiety is 32% compared to 14.1% in the general population.^4^, ^5^ People with mental illnesses have a 19% reduction in the odds of achieving abstinence when trying to quit,^6^ but are as motivated to quit as those without mental illness.^7^ These differences increase mortality in people with mental illness when compared to the general population resulting from cancer (mortality rate ratio: 1.92; 95% confidence interval [CI]: 1.91–1.94)^8^ and cardiovascular disease (mortality hazard ratio: 1.85; 95% CI: 1.53–2.24).^9^ Integrating cessation treatment into mental health settings could prevent 78,000 deaths in the next 80 years.^10^

People with mental illness may use smoking to try to alleviate symptoms, for example, using smoking to relax when they feel anxious;^11^ recent evidence suggests that this is counter‐productive, as smoking can in fact exacerbate and maintain mental health symptoms, and stopping smoking can improve mental health.^12^, ^13^, ^14^, ^15^ Qualitative studies suggest that although people with mental illness do report perceived benefits of smoking, they also accept evidence that smoking tobacco may harm mental health, and quitting might benefit mental health, and suggest that framing cessation as a treatment for mental health could motivate them to quit.^11^

A cochrane review of smoking cessation treatments for people with current and historical depression found that adding psychosocial mood management to usual smoking cessation treatment (e.g., nicotine replacement therapy) increased cessation rates when compared to usual smoking treatment alone (risk ratio: 1.47; 95% CI: 1.13–1.92). In the United Kingdom, people with depression/anxiety can access psychological therapy services, known as ‘Improving Access to Psychological Therapies’ (IAPTs), in which service users receive evidence‐based therapies to improve mood and well‐being. IAPT receives over 1.5 million referrals a year,^16^ and could offer smoking cessation treatment, but it currently does not. Integrating smoking cessation support within IAPT treatment for mental illness could improve physical and psychological outcomes for its service users. The World Health Organization recommends that countries integrate smoking cessation interventions into primary care services, such as IAPT.^17^

Qualitative studies embedded within randomized‐controlled trials (RCTs) provide the potential to gain new understandings of participant experiences of an intervention and inform the development of future interventions.^18^ We have recently codesigned a smoking cessation intervention with IAPT staff and service users, and are testing the intervention in a large, acceptability and feasibility pilot RCT.^11^, ^19^ We conducted interviews with trial participants to understand their experiences and views of the integrated smoking cessation intervention. We used the Capability, Opportunity and Motivation Model of Behaviour (COM‐B)^20^ to understand the:

1. Acceptability of the integrated smoking cessation treatment.
2. Views of psychological well‐being practitioners' (PWPs') ability to deliver the smoking cessation treatment.
3. Positive and negative impacts of smoking cessation treatment.

## 2 METHODS

This study was embedded within a feasibility RCT, prospectively registered on the ISRCTN registry (ISRCTN99531779).^19^ The data are available to bona fide researchers via successful application to the University of Bath.

We have followed COREQ reporting guidelines.^21^ Ethical approval for this study was received from the NHS Research Ethics Committee and the Health Research Authority on 19 March 2018.

### 2.1 Setting and participants

We conducted semi‐structured interviews with IAPT service users taking part in the ESCAPE Trial, involving four NHS trusts in the United Kingdom. Further details are described in the study protocol.^19^ In the ESCAPE Trial, intervention and control groups received usual care as part of IAPT (psychological therapy, such as Cognitive Behavioural Therapy (CBT), motivational interviewing etc.), lasting around 30–60 min. In the intervention group only, participants received smoking cessation support integrated within their IAPT sessions. The intervention included behavioural, psychological and pharmacological support adapted from the National Centre for Smoking Cessation and Training's (NCSCT) standard treatment programme.^22^

### 2.2 Recruitment procedure

We used a convenience sampling method. During trial follow‐up, we asked participants if they would like to take part in an interview about their experience in the study. Participants who had withdrawn from the study or did not complete follow‐ups were not approached for an interview. Informed consent was obtained verbally and recorded at the start of the interview; there was no written consent. The information sheet for the qualitative interviews was combined with the main trial information sheet.

To ensure confidentiality, with informed consent from participants, interviews were recorded using an encrypted digital voice recorder, transcribed and anonymized. Any identifying information in the transcripts was removed, but considering the risk of reidentification, researchers involved in the study were bound to confidentiality regulations set by the University of Bath and NHS. To further protect confidentiality, access to anonymized transcript data is restricted to only approved bona fide researchers after application to the University of Bath's Research Data Archive.

### 2.3 Sample size and selection criteria

For entry into the trial, participants fulfilled the eligibility criteria for IAPT and were daily tobacco smokers (see the trial protocol for details^19^). All trial participants were eligible for inclusion in the qualitative interviews, regardless of whether they had quit smoking or not during their participation in the trial; the final sample included both quitters and nonquitters.

We aimed to achieve strong information power.^23^ Information power was used based on the aim of the study being broad, sample specificity being moderate, use of applied theoretical frameworks (COM‐B), with moderate quality of dialogue, and a case and cross‐case analysis strategy.^23^ We agreed as a team that we reached strong information power at 36 participants.

#### 2.3.1 Interviews

Interviews were conducted between October 2018 and February 2021 over the telephone and lasted approximately 30–60 min. We used flexible interview schedules and open‐ended questioning (Supporting Information: Appendix S1). Interview schedules were modified as necessary throughout the course of the interviews to explore newly occurring concepts and experiences. Interviewers (K. S. and K. F. S.) kept notes to capture any relevant codes or concepts for analysis. Participants were not paid for the interview.

#### 2.3.2 Analysis

##### Analytic approach

Two researchers (K. S. and G. T.) conducted the analysis and held a critical realist perspective. Data were analysed using a framework approach to thematic analysis, following Braun and Clarke's^24^ method, with both deductive and inductive coding. This method was chosen as we aimed to compare the commonalities and differences in experiences of integrated treatment and relationships between experiences, both across cases and within individual cases.^25^ Deductive codes were informed by the COM‐B where appropriate; if constructs of the COM‐B were not identified in the data, they were not included in the final theme structure.^20^ Inductive codes were data‐driven and remained close to participants' language where possible. An example of data coded inductively and deductively can be found in Supporting Information: Appendix S2. The software used for data analysis were Microsoft Word and Excel.

##### Coding process and how themes were identified

One researcher (K. S.) read each transcript and listened to the audio recordings, followed by inductive line‐by‐line coding. After coding three transcripts, K. S. iteratively developed a data‐driven coding frame and sought feedback from the second researcher (G. T.). K. S. then grouped codes into categories, providing a working analytical framework that reflected the aims of the study, which were reviewed with G. T., and some inductive codes were added. K. S. then deductively coded the data based on the concepts from the COM‐B model, with some data being coded both inductively and deductively. K. S. then actively identified themes relating to study objectives, developed around the COM‐B model, which were reviewed and agreed with the wider team.

##### Reflexivity

Being aware of our own bias as researchers running and working on the ESCAPE trial, K. S. and G. T. kept notes and regularly checked in to discuss bias and the codes/themes being identified. Being aware of our own biases towards believing that the trial/therapy might succeed, we aimed to ensure that both positive and negative experiences and any deviant experiences are reflected in the results, such that themes are not necessarily all based on number. K. S. and G. T. both identify as females, K. S. has never smoked, G. T. is an ex‐smoker and K. S. and G. T. have not received mental health therapy in IAPT before.

### 2.4 Patient and public involvement

Before data collection, we consulted with people with lived experience of smoking and/or mental health problems and lay public members regarding the design of aims and interview schedules. After analysis, two people with lived experience of smoking and mental illness individually gave feedback on the final themes and quotes.

## 3 RESULTS

### 3.1 Participant characteristics

We invited 49 trial participants to take part in qualitative interviews. Thirteen declined to participate or did not answer recruitment calls, with the remaining 36 completing an interview at either the 3‐month or the 6‐month follow‐up, one person requested to be interviewed at both the 3‐ and 6‐month follow‐up time points. The mean age of the participants was 36.89 years (range: 20–65), 19/36 (53%) were female and the majority were White (92%) (Table 1). Most participants came from the Oxford Health NHS Foundation Trust (72%). Further demographic details are reported in Table 1, and the participant characteristics of in‐text quotations are available in Supporting Information: Appendix S3. Additional quotes relating to the study themes can be found in Supporting Information: Appendix S4.

**Table 1:** Clinical and demographic characteristics of the participants

	*n* (%)
Gender	
Female	19 (53)
Age (*M*/SD)	36.9 (11.5)
Ethnicity	
Other	31 (8)
White	33 (92)
Highest education	
A‐level equivalent	2 (5.6)
Apprenticeship	3 (8.3)
Degree	13 (36.1)
GCSE equivalent	5 (13.9)
Higher degree	6 (16.7)
Other vocational	7 (19.4)
Smoking status at Interview	
Quit (100% bioverified)	10 (27.8)
Smoking	26 (72.2)
Follow‐up interviewed at	
3 months	21 (58.3)
6 months	14 (38.9)
3 and 6 months	1 (2.8)
Pretreatment mental health (*M* [SD])	
Patient Health Questionaire‐9^26^	14.2 (6.2)
General Anxiety Disorder‐7^27^	12.4 (4.7)
Mental health at follow‐up (*M* [SD])	
Patient Health Questionaire‐9^26^	9.4 (5.7)
General Anxiety Disorder‐7^27^	8.5 (5.6)
Comorbid health conditions^a^	
Anxiety	13 (36.1)
Panic attacks	4 (11.1)
Obsessive‐compulsive disorder	2 (5.6)
Depression	2 (5.6)
Insomnia	1 (2.8)
None diagnosed	17 (47.2)

Abbreviation: GCSE, general certificate of secondary education (public exams in UK taken around age 16).

^a^ Participants could have had more than one comorbid health condition.

We identified four themes and nine subthemes (Table 2).

**Table 2:** Themes and subthemes

Theme (mapped onto the COM‐B framework)	Subtheme	Meets study aim
Theme 1: Psychological Capability Participants' psychological capability to accept and engage with integrated smoking cessation and IAPT treatment in the context of knowledge and stamina.	Subtheme 1.1 Integration supports mental health treatment and understanding	1, 3
	Subtheme 1.2: Knowledge and understanding of tobacco withdrawal	1, 3
Theme 2: Motivation Participants' reflective motivation and decisions to engage in integrated smoking cessation and IAPT treatment.	Subtheme 2.1: Openness to change when presenting to IAPT	1
	Subtheme 2.2: Curiosity and evaluation of previous quit attempts	1
Theme 3: Physical Opportunity Participants' physical opportunity to accept and engage in integrated smoking cessation and IAPT treatment. Physical opportunities are provided by environmental resources and time.	Subtheme 3:1 IAPT structure facilitates smoking cessation support	1
	Subtheme 3.2: Service‐level barriers to integrated treatment	1, 2
	Subtheme 3.3: Introducing an opportunity to quit	1
Theme 4: Social opportunity Participants' social opportunity to engage in integrated smoking cessation and IAPT treatment provided by interpersonal and social influences.	Subtheme 4.1: The value of the therapist–client alliance	2
	Subtheme 4.2: Holistic reflections on mental health and smoking experiences	2

Abbreviations: COM‐B, Capability, Opportunity and Motivation Model of Behaviour; IAPT, Improving Access to Psychological Therapies.

### 3.2 Theme 1: Psychological capability

#### 3.2.1 Subtheme 1.1: Integration supports mental health treatment and understanding

Participants described how they thought IAPT treatment models, such as CBT, ‘worked well’ with quitting smoking and how the integrated treatment benefitted mental health recovery.

*… I think it was a really good thing to have because now I think about it, quitting and also having the support with CBT is something that are probably something that go quite well together, hand in hand. Had I not quit smoking, I don't know whether the CBT would have had as much of an impact as it did or vice versa, so I think they worked really well together*. (Record 64)

Participants perceived that once the link between smoking and mental health was explained by the therapist, it ‘made sense’ that smoking and anxiety were related. Participants described how smoking acted as a ‘component’ of their anxiety, as a ‘temporary relief’, which then made them ‘feel worse’. Some participants described the physical effects of smoking, such as increased heart rate, relating this to anxiety in a ‘vicious cycle’.

#### 3.2.2 Subtheme 1.2: Knowledge and understanding of tobacco withdrawal

Some participants found that the integrated treatment affected their knowledge and capability to understand nicotine withdrawal symptoms, craving and anxiety. Participants described that they found it difficult to differentiate between withdrawal symptoms and mental health symptoms.

*The downside about quitting in conjunction I'd say for me anyway was that there is a big kind of flashing question mark, am I feeling rubbish because I'm quitting smoking, or is it because I'm depressed? Not being able to answer that it's a bit problematic. I've just come to the conclusion that there's no way of knowing, I have to think of them as two totally separate things*. (Record 50)

Some participants described craving and mental illness as two separate things: craving as a ‘niggling feeling’ and a want or need to go and smoke, whereas anxiety was described as overthinking, feeling panicky and nervous.

*The craving to me is like a niggling little feeling at the back of the mind, ‘I really want a cigarette, I really want this’. It's a monkey on my back that's basically telling me I need to go and have one. When it comes to mental illness, for me it's been over‐thinking things. Completely over‐thinking things. It's the anxiety in the way I sort of panic over different bits and pieces…because when I'm feeling anxious I relate smoking to relaxing. That's how it's been previously. I don't know if that makes sense?* (Record 89)

### 3.3 Theme 2: Motivation

#### 3.3.1 Subtheme 2.1: Openness to change when presenting to IAPT

When asked about their reasons for signing up to the integrated smoking cessation and IAPT treatment, participants reflected on their motivations and appeared to be open to change when first presenting to IAPT.

*Yeah, I thought it would be quite interesting to see whether or not it was possible to do the two at the same time, because obviously people tend to use smoking when they're stressed or worried about something, and if you're receiving treatment for anxiety and low moods it's kind of can you do both at the same time, or is it you have to focus on one?* (Record 51)

Participants described that they were pursuing a ‘change everything approach’, while they were presenting to the IAPT service. They were trying to change their mental state, their life, trying to ‘better themselves’. One participant described that at the time everything was changing so it was a ‘good a time as any’ to engage in both smoking cessation and mental health treatment. This suggests that participants accept integrated smoking cessation and IAPT treatment, and view presenting to mental health services as a good time to do both, although some participants did not find it helpful to address smoking and mental health together.

*…because you're focusing on trying to get your mental health better. At the start it seems you're less worried about quitting smoking*. (Record 65)

#### 3.3.2 Subtheme 2.2: Curiosity and evaluation of previous quit attempts

Many participants reflected on previous quit attempts, how some had been unsuccessful and described this as a motivation to try integrated treatment to see if it would help them successfully quit smoking. Participants described being curious to see whether quitting smoking would help their mental health and whether it would be possible to do both smoking cessation and mental health therapy at the same time. Many participants described how they had ‘tried a couple of avenues before which haven't worked’ and so thought ‘Why not try another to see if it helps …?’.

*I thought it was also an opportunity. I was kind of curious to see as well if the premise of quit smoking, less anxiety helps your mental health, I wanted to kind of see for myself. And I would work at it as well if it was something tangible that I could see as well. So, curiosity*. (Record 50)

### 3.4 Theme 3: Physical opportunity

#### 3.4.1 Subtheme 3:1 IAPT structure facilitates smoking cessation support

Participants described how having smoking cessation treatment integrated within IAPT treatment facilitated the opportunity to talk about any challenges when trying to quit. Participants said that having regular IAPT appointments offered a structure and opportunity to discuss smoking cessation regularly, which was ‘helpful’ and any issues did not have to ‘fester for too long’.

*There's nothing I didn't like about it. What I did like about it is the fact that we, we had the ability to talk about it every month, so during the sessions that I was talking to [Name] anyway, I knew that there was going to be a certain period of time where we would sit and go through any issues that there were and anything along those lines and I knew that there was support there if I needed it*. (Record 43)

The smoking cessation intervention fitted will within IAPT's delivery method of treatment programmes. Participants described how they found having integrated treatment over the phone ‘helpful’ and easier to fit into their daily lives.

#### 3.4.2 Subtheme 3.2: Service‐level barriers to integrated treatment

Some participants described how the length of time between appointments was too long and appointments were needed more frequently. Some participants described how there was a lot of content to fit into the appointments, which were often quite short. One participant described how they could sense their session was coming to an end as they felt their therapist was getting ‘stressed trying to fit everything into their appointment’.

*I've done two sessions with [organisation] and both times if I'm looking at a clock … I can tell when the speed is going to go up and their speaking, their rate of speech really rises the closer you get to the end. The amount of information that they want from you really drops as the time gets closer … I guess the only thing I would suggest is if they had a bit more time for calls…*. (Record 50)

One participant described how it felt ‘strange’ going from mental health to smoking cessation treatment. Another described how they felt that the smoking cessation treatment was an ‘add on’ and suggested scheduling smoking cessation treatment separately would allow them to give it ‘more emphasis, more thought and importance’.

*I didn't feel like the two… you know I said they felt different. I think it's useful to have them at the same time, but they were noticeably different in that delivering the CBT for the health anxiety is what this person does normally, and the intervention for smoking cessation isn't, and that felt kind of noticeable. But if that could be tackled, or maybe if they were merged a bit more, then perhaps it would be useful. I suppose I've got a really specific type of anxiety which if they were merged a bit better it could be really helpful. But not everybody has that*. (Record 41)

One participant also described their integrated treatment as ‘scripted’ and ‘unnatural’. Some participants described how setting clear objectives and an agenda with their therapist at the start of their session helped with the integration of the smoking cessation and IAPT treatment.

#### 3.4.3 Subtheme 3.3: Introducing an opportunity to quit

Many participants described how having the opportunity to access smoking cessation treatment prompted them to take part. One participant described how the smoking cessation support being available and offered whilst already seeking help for something else was important. They described how they accepted the offer in a ‘change of life scenario’ and decided to give the integrated treatment a go.

*I think it was the fact that I was seeking help for something else and this was an added benefit, so it was like I needed help for something and the offer was there to help me stop*. (Record 43)

### 3.5 Theme 4: Social opportunity

#### 3.5.1 Subtheme 4.1: The value of the therapist–client alliance

One participant described how the relationship with the PWP helped remove self‐blame around smoking as they could discuss their smoking as a coping mechanism in the context of their anxiety. Having the integrated treatment was described as a more positive and helpful experience than smoking cessation treatment alone.

Participants described how their PWPs encouraged them to make decisions and choices in their quit attempt, being guided in an encouraging and positive manner. Participants also emphasized how having to check in with their therapist regularly prompted them to remain abstinent from smoking as they did not want to let them down. Participants described how they were encouraged when they had had a ‘slip up’.

*She was very gentle, and I think she was very encouraging and very positive, but it was very much, I feel it was subtly getting me to make the decisions and getting me to make the choices, while acknowledging that these are all going to be good, she never actually said, ‘You must stop smoking’, it was always, ‘What benefits can you see from it? Can you think about why you don't stop, why you want to stop?’, it was very much guiding rather than leading. At the beginning there were hiccups, there was no judgement or condemnation, it was just, ‘These things happen, don't worry about it, it doesn't mean that you can't have another go’, and it was that, it was validating in a way that it was okay to slip up, but that doesn't negate having another go*. (Record 30)

#### 3.5.2 Subtheme 4.2: Holistic reflections on mental health and smoking experiences

Participants described how having the integrated treatment allowed them to share a whole picture of their mental health, and how their smoking was affected by their mental health and other stressors. Participants described how their therapist was ‘supportive but not like lecture‐y’ and their therapist tried to understand their smoking as part of their anxiety, ‘which no one's ever done before, so yeah, that was really helpful’. One participant said that the therapist understood what they were going through and were patient with them.

*Because she knew the difficulties I was going through as well, so rather than it being somebody talking to me from [service] and then somebody talking to me about my smoking, having two separate people, because it was the one person, she understood fully the struggles that life was bringing me, as well as trying to help me stop smoking, rather than feeling that…*. (Record 20)

Participants also felt like their smoking cessation support was tailored to them, compared to people who accessed an NHS stop smoking service, who felt like they were treated as a ‘generic smoker’, for example, being provided with information about the products available rather than identifying what would work for them as an individual.

## 4 DISCUSSION

### 4.1 Summary

We aimed to understand the experience of an integrated smoking cessation and mental health treatment among people with common mental illness. We found that generally, people with mental illness accepted integrated smoking cessation and mental health treatment, and had the psychological capability, motivation, physical and social opportunity to accept and engage with the integrated treatment. However, participants also faced several barriers in understanding tobacco withdrawal and at the service level. Participants described how PWPs had the interpersonal skills for delivering the smoking cessation intervention, but it sometimes seemed scripted or unnatural.

### 4.2 Strength and limitations

A strength of this study is that the findings are likely transferable to other primary care services, or similar services and populations, as the services involved in this study used nationally standardized treatments and service models, such as CBT, motivational interviewing and the NCSCT's standard treatment programme.^22^, ^28^ Most participants were White British, which was representative of the communities served by the NHS Trusts that we sampled,^16^ but the findings may not be generalizable to more ethnically diverse areas of the United Kingdom. However, our sample was broadly representative of general IAPT users in England, being mostly white, female and a younger age. Although the sample in this study is slightly older (average age 36.9 years) than the general IAPT service user population, those aged 18–24 years are most likely to access IAPT.^29^ Our sampling method could have introduced bias into the data, as we only sampled from those who completed follow‐ups, and it is possible that they could have had a more positive experience of the treatment than those who did not. Similarly, most participants completed interviews when they had completed treatment, so could have shown a recall bias where they reported mostly positive experiences of the intervention due to feeling more positive at the end of treatment, forgetting negative experiences.^30^

Use of a critical realist perspective allowed us to focus on understanding, instead of describing, social reality. Mental health treatment happens within a social reality as people and their actions influence the treatment pathways, and each is made up of, and influenced by, people's actions.^31^, ^32^, ^33^ A critical realist perspective assumes that human perceptions are accounts of reality, as what we observe is a social and subjective account of reality.^31^ Critical realism allows us to understand how and why interventions work within complex environments such as primary care mental health services.^31^ The benefits of a critical realist perspective are that we can understand the relationship between context (the setting of an intervention), mechanisms (things that cause change) and the outcome or experience of an intervention.^31^ Therefore, by using this perspective, we were able to understand how the experience of the smoking cessation intervention integrated within an IAPT service was influenced by the context in which it was experienced, and why or why not it was accepted in this environment.

### 4.3 Comparison with the existing literature

Integrated smoking cessation and mental health treatment was generally accepted, and participants had the capability to understand their smoking behaviour in the context of the tobacco withdrawal cycle and engage in treatment. These findings further those from our qualitative study of IAPT patients' views of integrated treatment, which found that IAPT patients accepted evidence that smoking may worsen their mental health and that quitting could improve their mental health.^11^ However, similar to other research, some participants described how they used smoking as a coping mechanism and prioritized their mental health treatment over quitting smoking.^34^, ^35^

Although most participants described their smoking using a CBT model and identified withdrawal as a component of their mental illness, some participants described withdrawal and mental illness as different experiences. This contrasts with the literature on the tobacco withdrawal cycle, which suggests that irritability and low mood from nicotine withdrawal are the same experience as mental illness.^36^, ^37^

This study found that an important factor for smoking cessation intervention uptake is having smoking cessation treatment available and offered in primary care services. This finding is in line with a systematic review that found that offering all smokers help to quit increased quit attempts, compared to telling them to quit.^38^ This study supports findings from our recent study, which suggests that IAPT services could be a suitable infrastructure for smoking cessation treatment, but there may be some service‐level barriers.^11^ Similar to a qualitative study of smoking cessation therapy for people with severe mental illness, we found that service users viewed PWPs as having good interpersonal skills to deliver smoking cessation interventions.^39^

Participants were motivated to accept and engage with the integrated smoking cessation and IAPT therapy, consistent with a systematic review indicating that people with mental illness are motivated to quit smoking.^7^ Participants stated that receiving smoking cessation treatment at the same time as mental health treatment was ‘a good a time as any’. These findings challenge health care professionals' views that quitting smoking at the same time is too much for people with mental illness.^35^, ^40^

Previous literature has suggested that altruism is an important motivator for participation in mental health trials.^41^, ^42^ The key motivators for engagement identified in the present study were participants' openness to change and curiosity regarding the potential impact of the combined treatment approach for smoking and their mental health. These motivations reflect a primary interest in the personal benefits of taking part rather than a desire to help others. These findings are consistent with the idea of ‘conditional altruism’,^43^, ^44^ which suggests that an interest in helping others may facilitate initial engagement; however, an expectation of some personal benefit is an important driver for enrolment and subsequent participation in trials.

### 4.4 Implications for research and practice

Integrating the NCSCT's standard treatment programme for smoking cessation^22^ into IAPT services is possible and accepted by people with common mental illness. Although there are financial considerations for IAPT to provide smoking cessation support on a larger scale, with funding required for training therapists, buying equipment, and so forth, it is important to remember that in the United Kimgdom, smoking costs our economy >£11bn per year.^45^ Given that participants reported that sometimes the intervention felt rushed or scripted, it could be that further intervention refinement is required, or that IAPT services should reduce PWP caseload to lengthen the session duration. In this trial, smoking was treated as a separate intervention programme; for truly integrated treatment, smoking should be addressed synonymously with other lifestyle behaviours. Future research should investigate how to achieve this.

## 5 CONCLUSIONS

People with common mental illness generally accepted integrated smoking cessation and mental health treatment. Smoking cessation treatment fits well within IAPT's structure; however, there are barriers to implementation related to resources. Reducing caseloads to allow for longer sessions with smokers would support implementation. Participants were open to change when first presenting to IAPT and motivated by curiosity to see whether quitting smoking would help their mental health. Participants viewed PWPs as having good interpersonal skills to deliver smoking cessation intervention.

## AUTHOR CONTRIBUTIONS

Ms Katherine Sawyer led on data curation, investigation, project administration, data analysis, writing and editing the manuscript. Dr Kim Fredman Stein contributed to data curation, investigation, project administration and data analysis. Dr Pamela Jacobsen supervised the analysis and investigation, and contributed to writing and editing the manuscript. Dr Tom P. Freeman supervised the analysis and investigation, and contributed to writing and editing the manuscript. Dr Anna K. M. Blackwell made a substantial contribution to interpretation of data, and writing and editing the manuscript. Prof Chris Metcalfe contributed to study conceptualization, methodology, analysis, investigation, writing and editing of the manuscript and funding acquisition. Prof David Kessler contributed to study conceptualization, methodology, analysis, investigation, writing and editing of the manuscript and funding acquisition. Prof Marcus Munafò supervised study conceptualization, investigation and funding acquisition, and contributed to the study methodology, analysis, writing and editing of the manuscript and project administration. Prof Paul Aveyard supervised study conceptualization, analysis, investigation, writing and editing of the manuscript and funding acquisition, and contributed to methodology and project administration. Dr Gemma Taylor led on study conceptualization, methodology and funding acquisition, contributed to analysis, investigation, administration, data curation, editing the manuscript and supervision of analysis.

## CONFLICTS OF INTEREST

Marcus Munafò and Gemma Taylor previously received funding from Pfizer, who manufacture smoking cessation products, for research unrelated to this study. No other authors have any potential conflict of interest to declare.

## ETHICS STATEMENT

Ethics approval for this study was received from the NHS Research Ethics Committee and the Health Research Authority on 19 March 2018, IRAS ID 239339.

## Supporting information

Supporting information.

## Data Availability

Anonymized transcript data are available via application to the University of Bath Research Data Archive.
