# Supplementary material for: Acceptability of integrating smoking cessation treatment into routine care for people with mental illness: A qualitative study
Source: Health Expect. 2022 Oct 12;26(1):108–18. doi: 10.1111/hex.13580 (PMC9854288; doi:10.1111/hex.13580)
Supplement: Supplementary file 1 — Supporting information. [file HEX-26--s001.docx]

SUPPORTING INFORMATION 1-4

Table of Contents

[APPENDIX S1: INTERVIEW SCHEDULE FOR STUDY PARTICIPANTS 2](#_Toc110243995)

[APPENDIX S2: EXAMPLES OF DATA CODED INDUCTIVELY AND DEDUCTIVELY 3](#_Toc110243996)

[APPENDIX S3: PARTICIPANT CHARACTERISTICS 4](#_Toc110243997)

[APPENDIX S4: ADDITIONAL QUOTES 5](#_Toc110243998)

# APPENDIX S1: INTERVIEW SCHEDULE FOR STUDY PARTICIPANTS

**Questions about recruitment/signup**

Can you tell me a little about how you found out about the study?

Can you tell me a little about why you decided to sign up?

Can you tell me a little about the treatment you received?

**Questions for about quitting in intervention arm**

How did you get on with your quit attempt?

What did you find difficult?

What did you find easy?

Was there anything you would change?

What did you like/not like about the smoking cessation support you received?

What were the barriers/facilitators to quitting smoking?

How did you feel when you were trying to quit/quitting/relapsing?

**Questions about quitting in control arm**

Did you access your local stop smoking services?

How did you find the referral processes?

Did you try and quit smoking?

How did you get on with your quit attempt?

What did you find difficult?

What did you find easy?

Was there anything you would change?

What did you like/not like about the smoking cessation support you received?

What were the barriers/facilitators to quitting smoking?

How did you feel when you were trying to quit/quitting/relapsing?

**Questions about trial procedures**

How did you find being randomly assigned to receive help to quit?

Did you feel that your therapist was supportive in helping you to quit?

What did you think about easy/difficult about getting your medication?

What did you like/not like about the smoking cessation support from your therapist?

Did you receive any smoking cessation information via mail/email/handout?

What did you think about that information?

Would you have changed anything?

# APPENDIX S2: EXAMPLES OF DATA CODED INDUCTIVELY AND DEDUCTIVELY

Deductive: Reflective Motivation

Inductive: Curiosity, interested in finding out it quitting smoking helps their mental health, interested to see if you can do both treatments at the same time.

*“I thought it was also an opportunity. I was kind of curious to see as well if the premise of quit smoking, less anxiety helps your mental health, I wanted to kind of see for myself. And I would work at it as well if it was something tangible that I could see as well. So, curiosity.” (Record 50)*

*“Yeah, I thought it would be quite interesting to see whether or not it was possible to do the two at the same time, because obviously people tend to use smoking when they're stressed or worried about something, and if you're receiving treatment for anxiety and low moods it's kind of can you do both at the same time, or is it you have to focus on one?” (Record 51)*

Deductive: Social Opportunity

Inductive: Therapist-client alliance and integrated treatment helps to remove self-“blame” around smoking, and foster “opening-up”, allows client to share “whole picture” of their mental health and smoking experiences.

*“she was very gentle, and I think she was very encouraging and very positive, but it was very much, I feel it was subtly getting me to make the decisions and getting me to make the choices, while acknowledging that these are all going to be good, she never actually said, “You must stop smoking”, it was always, “What benefits can you see from it? Can you think about why you don’t stop, why you want to stop?”, it was very much guiding rather than leading. At the beginning there were hiccups, there was no judgement or condemnation, it was just, “These things happen, don’t worry about it, it doesn’t mean that you can't have another go”, and it was that, it was validating in a way that it was okay to slip up, but that doesn’t negate having another go” (Record 30).*

*“Because she knew the difficulties I was going through as well, so rather than it being somebody talking to me from [Organisation] and then somebody talking to me about my smoking, having two separate people, because it was the one person, she understood fully the struggles that life was bringing me, as well as trying to help me stop smoking, rather than feeling that...*

*I think I personally feel if it had been somebody separate, they wouldn’t have understood and been quite so patient with me at the fact that I wasn’t managing it, because they wouldn’t have had the whole picture, and I wouldn’t have felt comfortable talking to two people about what was going on in my life and why I was where I was, having the difficulties. But to do it all with one person, I felt it was a better way for me to try to do it. Even though it failed, I don’t think I’d have coped with talking to two people.” (Record 20)*

*“We had that professional relationship, I saw him as a mentor and somebody to kind of guide me because at that time, I felt I was lost and I needed someone to hold my hand and guide me on the path essentially for myself, kind of like redemption really and I feel that yeah, he has successfully achieved that and I am in a better place now than I was before, so I’d say I did accomplish my goals” (Record 92).*

| **Unique ID** | **Trial Arm** | **Smoking Status** | **Gender** | **Age** |
| --- | --- | --- | --- | --- |
| Record 7 | Intervention | Smoking | Male | 26 |
| Record 8 | Control | Smoking | Male | 30 |
| Record 13 | Control | Smoking | Female | 33 |
| Record 20 | Intervention | Smoking | Female | 42 |
| Record 22 | Intervention | Quit | Female | 35 |
| Record 30 | Intervention | Quit | Female | 51 |
| Record 34 | Control | Smoking | Male | 33 |
| Record 41 | Intervention | Quit | Female | 34 |
| Record 43 | Intervention | Quit | Male | 54 |
| Record 48 | Intervention | Smoking | Female | 28 |
| Record 50 | Intervention | Quit | Male | 60 |
| Record 51 | Control | Smoking | Female | 31 |
| Record 52 | Intervention | Smoking | Female | 40 |
| Record 64 | Intervention | Quit | Female | 23 |
| Record 65 | Intervention | Smoking | Male | 42 |
| Record 78 | Intervention | Smoking | Female | 65 |
| Record 89 | Control | Smoking | Male | 42 |
| Record 92 | Intervention | Smoking | Male | 24 |
| Record 95 | Control | Smoking | Female | 22 |
| Record 97 | Intervention | Quit | Male | 35 |

# APPENDIX S3: PARTICIPANT CHARACTERISTICS

**Table 1 Participant characteristics of in text quotations.**

# APPENDIX S4: ADDITIONAL QUOTES

**Theme 1: Psychological Capability**

*Subtheme 1.1: Integration can support mental health treatment and understanding*

*“Being on this trial, stopped smoking, it has worked, it’s broken that circle so I don’t feel as stressed anymore, I feel much calmer in my everyday life, it’s much more enjoyable” (Record 97).*

*“Yeah, it did because I was, it gave me something that straight off the bat it was, well I haven’t had a cigarette now in four days. Not a personal best but it was a case of I’ve been smoking so long now, four days is actually really good; I want a cigarette but no, why should I smoke? I don’t need to smoke, I don’t have any reason to, that’s going good for me. So, by the time I was getting to work, I already had a positive outlook for the start of the day, because I’d already achieved something for the past three days, so let’s do it for the fourth and then let’s do it for the fifth and the sixth and yeah, for myself it was just a boost because I’d actually accomplished something and was still accomplishing it” (Record 07).*

*“Yeah, I kind of knew that [SMOKING] was like a coping mechanism that, in the short term, seemed to help but in the long term then I’d worry about. And I felt like I’d let myself down if I tried to give up and then I couldn’t. So, then when we unpicked it in CBT, it made sense that actually, it was a worry behaviour, and it wasn’t something that I had to do, and it was making me feel worse.” (Record 22)*

*Subtheme 1.2: Knowledge and understanding of tobacco withdrawal*

“….it’s [mental health treatment] primarily about thought processing for me, and dealing with the way I feel and working strategies together and how to address these. Whilst the smoking cessation is more to do with say how to address temptation and what to do in times when you feel overwhelmed by the feeling that you need to have that smoke. There were some similarities, but I wouldn’t say it was exactly the same” (Record 92).

**Theme 2: Motivation**

*Subtheme 2.1: Openness to change when presenting to IAPT*

*“I always wanted to. But I think at the time I was trying to better myself, so at the time I thought we'll give it a go at the same time, why not sort of thing” (Record 48)*

*Subtheme 2.2: Curiosity and evaluation of previous quit attempts*

*“Yeah, I thought it would be quite interesting to see whether or not it was possible to do the two at the same time, because obviously people tend to use smoking when they're stressed or worried about something, and if you're receiving treatment for anxiety and low moods it's kind of can you do both at the same time, or is it you have to focus on one?” (Record 51).*

*“Yeah. I’ve wanted to stop smoking for a long time. I’ve tried many times in the past and it’s always failed so I thought maybe at the time I was having help and support to try and help change the way I was thinking, I thought that might be a good time” (Record 52).*

*“I've really struggled with the isolation, that side of things, the COVID, it's really hard hit me. I'm one of these people who is quite an extrovert, I like to go out, I like to do stuff, I'm always busy, and it has really impacted me the fact that I can't go out, I can't go off to restaurants, I can't go and meet friends. That has really affected me, and that's also driven up the amount of smoking I'm doing” (Record 89).*

**Theme 3: Physical Opportunity**

*Subtheme 3:* *IAPT structure facilitates smoking cessation support*

*“I think that the provision of nicotine replacement so easily is really, really helpful. It's not something that I ended up needing in the end, but it's one less barrier. I'm the type of person I'm happy to go out and buy nicotine replacement therapy, it's actually going out and doing it, so having it delivered to the house was really helpful. And on a scheduled basis as well where I didn't have to think about it, it's just there, that's very helpful” (Record 41).*

*Subtheme 3.2: Service level barriers to integrated treatment.*

*“But I think probably the biggest difficulty with a programme like this done in conjunction with [organisation], is basically the length of time between five/ten minute chats. When you're going through withdrawal that's really not sufficient. So if there were something that could kind of fill that gap, that would probably be really good” (Record 50).*

*“Having to wait a fortnight for the therapy and even the smoking cessation, you need a bit more frequent, it doesn’t have to be as long, an hour it didn’t have to be but just a much shorter time would have been … and more frequently, would have been more helpful than like an hour or two hours every fortnight or every month, you know? I’ve got another Facetime one tomorrow. Slowly but surely” (Record 78).*

*“Yeah, from the moment I picked up the phone to trying to speak to someone, for the initial sort of starting point, that took quite long, that took about a month and a half to get that started. But as soon as it did start, it was excellent. [Name] did he explain to me why, where he works, they only have two or three people that do the anxiety and smoking, that’s why it took so long, he said” (Record 97).*

*“I found that quite easy because the guy would generally say, “in a couple of minutes, we’re going to switch over” and then he used to get into conversation with my anxiety, to then automatically roll it into the smoking conversation. I didn’t really notice and before you know it, the time’s up” (Record 97).*

*Subtheme 3.3: Introducing an opportunity to quit*

*“I think it was the fact that I was seeking help for something else and this was an added benefit, so it was like I needed help for something and the offer was there to help me stop. Even though it was, how do I word this? Yeah, the offer was there, so I decided that I was going to take the offer as well and it was a change of life scenario, so to speak” (Record 43).*

**Theme 4: Social Opportunity**

*Subtheme 4.1: The value of the therapist-client alliance*

*“So, I suppose an advantage of having them together was every week someone’s checking in on you, with your worry behaviour but also with your not smoking... it’s not just all about not smoking, it’s about the whole worry and the whole anxiety and not blaming yourself, I think that helped as well, it’s like you can just go and talk about what’s going wrong and not feel bad about it. I felt like that made it easier to give up, it’s kind of stop blaming yourself, this is a coping mechanism, lots of people do it, lots of people with anxiety smoke, and then just like, OK, what can we do to help? So, I felt like doing it within CBT rather than just on its own made it a more positive, helpful experience” (Record 22).*
